# Impact of the 24-h ultramarathon race on homocysteine, oxidized low-density lipoprotein, and paraoxonase 1 levels in professional runners

**DOI:** 10.1371/journal.pone.0192392

**Published:** 2018-02-02

**Authors:** Serena Benedetti, Simona Catalani, Federica Peda, Francesca Luchetti, Roberto Citarella, Serafina Battistelli

**Affiliations:** 1 Department of Biomolecular Sciences, Section of Clinical Biochemistry and Molecular Genetics, University of Urbino “Carlo Bo”, Urbino, Italy; 2 Department of Biomolecular Sciences, Section of Pharmacology and Pharmacognosy, University of Urbino “Carlo Bo”, Urbino, Italy; 3 Centre of Rehabilitation Therapy, Reggio Emilia, Italy; Medical University Innsbruck, AUSTRIA

## Abstract

The impact of the 24-h ultramarathon race on homocysteine (Hcy) and oxidized low-density lipoprotein (oxLDL) levels, two well-recognized cardiovascular risk factors, has not been deeply investigated. Similarly, no information exists on paraoxonase 1 (PON1), an antioxidant enzyme associated with high-density lipoproteins, which may detoxify oxLDL and Hcy-thiolactone, hence preventing their proatherogenic action. Taking this into account, a competitive 24-h ultramarathon race was organized in Reggio-Emilia (Italy) recruiting professional runners (n = 14) from the Italian Ultramarathon and Trail Association. Blood samples were collected from each participant before, during (14 h), and immediately after (24 h) the competition, thus to monitor the serum changes in Hcy, oxLDL, and PON1 levels, as well as other oxidative stress-related parameters, namely reactive oxygen metabolites (ROM) and total antioxidant capacity (PAT). As a result, a significant PON1 increase was recorded after 14 h of racing that persisted until the end of the performance. The same trend was observed for PAT values, which positively correlated to PON1 levels (R = 0.643, P<0.001). Hcy, oxLDL, and ROM remained almost unchanged throughout the competition. In conclusion, the present study suggested a protective role of PON1 in sustaining the antioxidant defense system and contrasting lipoprotein oxidative modifications over the 24-h race, and did not specifically evidence either Hcy or oxLDL accumulation in such challenging sporting events.

## Introduction

The 24-h ultramarathon race is one of the most demanding competitive sports in terms of muscular and physiological exertion, and is characterized by the release of pro-inflammatory factors and generation of reactive oxygen species (ROS) which can lead to oxidative stress [1–4]. The main source of ROS is the leakage of electrons from the mitochondrial electron transport chain during ATP synthesis by oxidative phosphorylation [5]; however, other important sources include activated neutrophils and macrophages recruited to repair damaged tissues [6]. Indeed, exercise-induced microinjuries to muscles and other tissues [1, 2, 7, 8] lead to increased migration of white blood cells to the sites of injury and induction of acute phase inflammatory reactions [9, 10].

In this context, abnormal ranges of cardiac markers as well as an increased risk for adverse cardiovascular events have been reported in ultraendurance athletes [11–13]. Homocysteine (Hcy) and oxidized low-density lipoproteins (oxLDL) are two well-recognized risk factors related to oxidative stress involved in cardiovascular pathology and atherosclerotic plaque development [14–16]; nevertheless, the effect of the 24-h ultramarathon race on Hcy and oxLDL levels has not been deeply investigated. Similarly, no information exists on the protective role of paraoxonase 1 (PON1), an antioxidant enzyme associated with high density lipoproteins (HDL), which may detoxify oxLDL and Hcy-thiolactone, hence preventing their proatherogenic action [17, 18]. In fact, PON1 esterase activity may hydrolyze oxidized lipids present in lipoproteins as well as in atherosclerotic lesions, while PON1 lactonase activity may hydrolyze Hcy-thiolactone, a highly reactive intermediate that can contribute to atherogenesis by inducing endothelial cell damage either by toxicity itself or by homocysteinylation of cell proteins [17, 18].

On the basis of the above considerations, aim of the present investigation was to evaluate the impact of the 24-h ultramarathon race on different biochemical and metabolic parameters with special emphasis on Hcy, oxLDL and PON1, in order to derive the possible effect of 24-h running as cardiovascular risk factor. Other oxidative stress-related parameters, namely reactive oxygen metabolites (ROM) and total antioxidant capacity (PAT), as well as routinely available blood biomarkers (organ function and damage, inflammation, metabolic profile), were also assessed.

## Methods

### Subjects

A competitive 24-h ultramarathon race was organized in Reggio-Emilia (Italy) on March 12, 2016, in collaboration with the Italian Ultramarathon and Trail Association (IUTA), the Centre of Rehabilitation Therapy of Reggio-Emilia (Italy), and the University of Urbino (Italy). The race selectively involved professional runners from IUTA national team, who were interested in participating in the study. No specific exclusion criteria were defined; indeed, all the runners were healthy (no history of cardiovascular diseases), endurance-trained athletes fully familiarized with ultramarathon running (approximately three 24-h races per year). After being advised about the experimental procedures, 14 out 20 athletes approached (M = 7, F = 7, age 30–58 years, body weight 48–78 kg, height 158–180 cm) gave their written informed consent to participate in the study. Protocols were submitted to, and approved by, the Institutional Review Board of IUTA, in accordance with the Declaration of Helsinki.

The race was performed on a 1000 m certified flat trail (58 m above sea level); it started at 10.00 a.m. and ended at 10.00 a.m. of the following day. Temperature at the competition site varied from 7°C to 15°C; humidity ranged from 51% to 93%. The participants were allowed to rest and ingest liquids and food freely, properly recorded (S1 File). Liquids consisted mainly of water, soft drinks, and sport beverages, while food mostly included fruits, pasta, cookies, and energy bars.

### Blood sampling

Venous blood samples were collected from each participant 3 h before the race (after an overnight fasting) (pre-race), after 14 h from the beginning of the race (intra-race), and immediately after the race (post-race). Samples (collected both in sterile EDTA-containing tubes and in serum separator tubes) were immediately transported at controlled temperature to the clinical laboratories for the subsequent biochemical analysis.

### Biochemical analyses

Routine blood analysis were performed at a local laboratory sited in Reggio-Emilia (Italy) by automated analyzers (Sysmex XT1800i, Dasit; Olympus AU480, Beckman Coulter; Access 2, Beckman Coulter). Basic blood count included red blood cells (RBC), hematocrit (Ht), hemoglobin (Hb), platelets (Plt), and white blood cells (WBC). Leukocyte subpopulations and high-sensitive C-reactive protein (CRP) were also assessed. Uric acid, creatinine, blood urea nitrogen (BUN), glomerular filtration rate (MDRD), gamma-glutamyl transferase (GGT), aspartate aminotransferase (AST), alanine aminotransferase (ALT), creatine phosphokinase (CPK), cardiac creatine phosphokinase (CPK-MB), and troponin-I were evaluated as markers of organ function and damage. Regarding the metabolic profile, total proteins, glucose, triglycerides (TG), total cholesterol (TC), low-density lipoproteins (LDL), and high-density lipoproteins (HDL) were determined. The lipid ratios TC/HDL, LDL/HDL, and TG/HDL were also calculated. Other serum parameters included homocysteine (Hcy) and related parameters (vitamin B6, vitamin B12, and folates).

Oxidative stress evaluations were performed at the University of Urbino (Section of Clinical Biochemistry and Molecular Genetics). Serum samples were obtained by blood centrifugation at 2500 rpm for 10 min. at 4°C and then stored at -80°C until used. Reactive oxygen metabolites (ROM, principally hydroperoxides) and total antioxidant capacity (PAT) were assessed spectrophotometrically in serum samples by ROM test and PAT test, respectively (H&D, Parma, Italy). As reported by the Manufacturer, ROM reference values in the healthy population range from 20 to 24 mg/dl H_2_O_2,_ while PAT levels from 3080 to 3920 μmol/l vitamin C. Oxidized LDL (oxLDL) were evaluated by a specific immunoassay from Biomedica (Vienna, Austria) (detection limit 0.05 μg/ml, CV intra-assay <10%), while paraoxonase 1 (PON1) by an immunoassay from Elabscience Biotechnology (WuHan, P.R.C.) (detection limit 0.094 ng/ml, CV intra-assay <10%). To obtain internal reference values for oxLDL and PON1 levels, serum samples were collected after informed consent from 11 healthy volunteers (M = 6, F = 5, age 37±12 years) attending the Blood Transfusion Centre of the local Hospital. Values ≤132 μg/dl were obtained for oxLDL levels, while values ranging from 14 to 38 μg/dl were evidenced for PON1.

### Statistics

Data are presented as mean ± standard deviation (SD). Repeated-measures of analysis of variance (ANOVA) were used to analyze time-course changes, followed by the Bonferroni post-hoc test (SPSS software, IBM). T-test for paired or unpaired data was also applied as appropriate. Linear regression analysis was used to assess relationships between selected variables. Statistical significance was defined as P<0.05.

## Results and discussion

The present investigation specifically involved professional athletes from IUTA national team running a 24-h ultramarathon race. Due to this selective inclusion criterion, the number of participants was limited and not based on sample-size calculation. All the competitors reached the 14-h distance, running 117.1±14.3 km with a mean speed equal to 8.4±1.1 km/h. However, out of 14 athletes, only 9 runners (M = 5, F = 4) completed the 24-h race and could be included in the study. Their baseline characteristics and running performance data are summarized in Table 1. For each athlete, the highest running velocity was attained at the first stage of the race, i.e. after 14 h from the beginning of the competition; then it decreased to reach a significantly lower level over the whole distance covered. There were marked differences in individual race performance measures; however, running velocity was not significantly related to age, sex, body mass, or BMI. Noteworthy, the 24-h speed appeared to be positively correlated with VO_2max_ (R = 0.705, p = 0.05), thus confirming previous evidence on the importance of a high VO_2max_ for performance in ultraendurance events [1, 19].

**Table 1:** Baseline characteristics and ultramarathon records of the runners which completed the race (n = 9).

	Males (n = 5)		Females (n = 4)	
Characteristics	Mean±SD	Min-Max	Mean±SD	Min-Max
Age (years)	41±11	30–58	45±9	33–55
Height (cm)	173±6	165–180	163±2*	161–164
Weight (kg)	71.6±4.2	67–78	57.1±2.7*	54.7–60.0
BMI	23.9±1.3	22.3–25.8	21.5±1.5*	20.1–23.2
HR (bpm)	50±1	48–51	61±7*	53–65
SBP (mmHg)	135±13	123–153	113±9*	103–121
DBP (mmHg)	78±9	65–87	72±9	62–78
VO_2max_ (ml O_2_/kg/min)	50.4±13.1	36.7–66.5	43.3±14.6	35.0–50.1
**Ultramarathon records**				
14-h distance (km)	122.8±5.2	117.5–128.7	119±16	101.3–130.7
14-h mean speed (km/h)	8.7±0.4	8.3–9.0	8.4±1.1	7.2–9.2
24-h distance (km)	184.4±33.7	146.9–237.0	167.8±44.2	117.5–200.6
24-h mean speed (km/h)	8.1±1.4^#^	6.2–9.8	7.2±1.7^#^	5.3–8.5

*p<0.05 vs. males (T-test for unpaired data)

^#^p<0.05 vs. 14-h mean speed (T-test for paired data). Abbreviations: BMI, body mass index; DBP, diastolic blood pressure; HR, heart rate; SBP, systolic blood pressure; VO_2max_, maximum oxygen consumption.

Overall, the 24-h race was associated with severe muscle damage, as already observed in similar competitions [1, 2, 7, 20, 21]. The strong increment of serum CPK, ALT and AST levels was significant already after 14 h of racing, and a further increase was recorded at the end of performance (Table 2). In this context, increases in ALT and AST did not specifically indicate liver cell damage but rather muscle cell damage; indeed, fairly stable serum GGT levels were observed during the race, suggesting minor hepatic injury, as previously reported [1, 7, 20]. In accord with Waskiewicz et al. [1], a positive correlation was found between the distance covered at 14 and 24 h and the corresponding levels of CPK (R = 0.645, P = 0.024), ALT (R = 0.872, P<0.001), and AST (R = 0.786, p = 0.002). Other authors [21] found a negative correlation between the 24-h distance and CPK levels, and suggested that the volume of the effort might not necessarily be the major determinant of CPK increase.

**Table 2:** Ultramarathon-induced changes in basic blood count, leukocyte subpopulations, markers of organ function and damage, and inflammation. Data, obtained from athletes which completed the race (n = 9), are corrected for Ht^§^ and expressed as mean±SD.

Basic blood count	Unit	Reference	Pre-race			Intra-race			Post-race		
RBC	x10^6^/μl	4–5.9	4.90	±	0.45	4.73	±	0.51	4.70	±	0.67
Hb	g/dl	13–17.5	14.61	±	1.15	14.18	±	1.32	14.04	±	1.76
Ht	%	39–55	43.07	±	2.91	40.56	±	3.50*	40.17	±	4.77*
Plt	x10^3^/μl	140–450	236.7	±	47.8	279.7	±	41.9*	256.8	±	50.9*
WBC	x10^3^/μl	3–10.8	6.56	±	1.63	16.77	±	4.69*	14.82	±	6.09*
**Leukocyte subpopulations**											
Neutrophils	x10^3^/μl	1.8–7	3.49	±	1.49	13.58	±	4.20*	11.67	±	5.61*
Lymphocytes	x10^3^/μl	1–4.8	2.19	±	0.74	1.57	±	0.57	1.68	±	0.81
Monocytes	x10^3^/μl	0–1.1	0.64	±	0.22	1.60	±	0.56*	1.40	±	0.62*
Eosinophils	x10^3^/μl	0–0.7	0.24	±	0.12	0.02	±	0.04*	0.04	±	0.07*
Basophils	x10^3^/μl	0–0.2	0.02	±	0.04	0.01	±	0.03	0.00	±	0.00
**Organ function and damage**											
Uric acid	mg/dl	3.5–7.2	4.50	±	0.77	5.12	±	1.24*	4.71	±	1.16
Creatinine	mg/dl	0.7–1.3	0.79	±	0.12	0.97	±	0.17*	0.91	±	0.19
BUN	mg/dl	10–50	28.33	±	5.07	63.00	±	18.14*	58.19	±	25.46*
MDRD	ml/min/1,73mq	>60	97.22	±	17.15	87.13	±	14.20	95.76	±	17.14
GGT	U/l	<55	25.22	±	17.79	25.55	±	17.07	23.89	±	15.90
AST	U/l	7–45	25.00	±	6.48	87.07	±	14.13*	201.50	±	73.52*^#^
ALT	U/l	7–45	20.71	±	6.55	33.07	±	4.33*	56.11	±	11.20*^#^
CPK	U/l	<171	114.43	±	39.69	2132.31	±	720.05*	4924.85	±	2829.76*^#^
CPK-MB	ng/ml	0.6–6.3	4.23	±	2.04	67.40	±	29.39*	149.52	±	87.84*^#^
Troponin-I	ng/ml	<0.04	0.02	±	0.03	0.13	±	0.13*	0.12	±	0.10*
**Inflammation**											
CRP	mg/l	<1	0.10	±	0.12	0.51	±	0.35*	3.12	±	1.46*^#^

*Significantly different from pre-race values, p<0.05

^#^significantly different from intra-race values, p<0.05.

^§^Ht levels significantly decreased after 14 and 24 h of racing as compared to baseline values, indicating the presence of hemodilution throughout the competition. For this reason, all the biochemical parameters were corrected for changes in plasma volume, as previously indicated [7]. Abbreviations: ALT, alanine aminotransferase; AST, aspartate aminotransferase; BUN, blood urea nitrogen; CPK, creatine phosphokinase; CPK-MB, cardiac creatine phosphokinase; CRP, C-reactive protein; GGT, gamma-glutamyl transferase; Hb, hemoglobin; Ht, hematocrit; MDRD, glomerular filtration rate; Plt, platelets; RBC, red blood cells; WBC, white blood cells.

The increment of urea and creatinine is another common finding among ultramarathon runners [21, 22]. It might reflect increased protein catabolism in muscle cells following strenuous physical exercise, and/or a decrease in renal blood flow and glomerular filtration rate due to kidney injury. Herein, MDRD, estimating the glomerular filtration rate, remained almost unchanged throughout the competition (Table 2); therefore, acid uric, BUN and creatinine increments might be explained by the increased catabolic state of skeletal muscle, as previously suggested [21, 22].

Regarding the assessment of myocardial injury, elevations in CPK-MB and troponin-I levels were observed (Table 2). The increment of CPK-MB might lack specificity for cardiac damage, as distance runners have increased CPK-MB in their skeletal muscle as well. On the contrary, the increment of troponin-I, a sensitive and specific cardiac marker, sustained the evidence for exercise-induced cardiac stress throughout the competition, as already reported [21, 22]. CPK-MB and troponin-I positively correlated to each other (R = 0.530, P = 0.013); at the same time, a positive and significant correlation was found between the running distance and CPK-MB (R = 0.820, P = 0.001) but not between distance and troponin-I levels, suggesting that other factors than distance may influence cardiac troponin elevations, as extensively described by Gresslien and Agewall [23].

In response to tissue damage, a significant increase in total WBC count was recorded after 14 h of racing, which persisted until the end of the performance (Table 2); the major components were neutrophils and monocytes, as previously observed [1, 2, 21]. An induction of acute-phase inflammatory reactions also occurred, as demonstrated by the increment of hepatocyte-derived CRP [7, 24]. In agreement with Waskiewicz et al. [1], a positive correlation was found between the distance covered and CRP levels (R = 0.636, P = 0.011); at the same time, CRP positively correlated to CPK (R = 0.469, P = 0.032), AST (R = 0.579, P = 0.006), ALT (R = 0.671, P<0.001), and CPK-MB (R = 0.449, P = 0.041), sustaining the close connection between tissue damage and inflammation [7].

Besides comparing changes in tissue function and injury variables, the metabolic responses to the 24-h race were also taken into consideration (Table 3). Confirming previous evidence on similar ultraendurance events [1, 7, 22], no decrements in blood glucose concentration were found; rather, glucose levels significantly increased throughout the race, which implied adequate carbohydrate supply during the performance (see S1 File for information on individual food and liquid intake). On the other hand, the competition had a major impact on fat metabolism; indeed, TG levels significantly decreased at the end of the event, reflecting the use of fatty acids as fuels, in accord with previous observations [1, 2, 25]. The decrement in TG was accompanied by a significant decline in TC and LDL levels, and a significant rise in HDL. The same findings were reported by Waskiewicz et al. [1], which specifically investigated the acute metabolic responses to a 24-h competition.

**Table 3:** Ultramarathon-induced changes in blood parameters related to metabolic profile, oxidative stress, and Hcy metabolism. Data, obtained from athletes which completed the race (n = 9), are corrected for Ht^§^ and expressed as mean±SD.

Metabolic profile	Unit	Reference	Pre-race			Intra-race			Post-race		
Total proteins	g/dl	6–8.3	6.93	±	0.36	7.39	±	0.43	7.24	±	0.41
Glucose	mg/dl	60–110	98.22	±	9.08	130.53	±	20.43*	111.27	±	19.83#
TG	mg/dl	<150	79.89	±	25.75	95.00	±	18.89	59.10	±	13.07#
TC	mg/dl	<200	192.78	±	48.45	195.42	±	48.66	179.18	±	50.76#
LDL	mg/dl	<115	113.11	±	33.98	110.31	±	34.77	97.10	±	36.14*#
HDL	mg/dl	>45	65.44	±	13.12	72.72	±	14.38*	74.21	±	14.68*
TC/HDL	-	<5	2.95	±	0.49	2.69	±	0.43*	2.41	±	0.44*#
LDL/HDL	-	<2	1.74	±	0.42	1.52	±	0.38*	1.31	±	0.39*#
TG/HDL	-	1–2	1.27	±	0.48	1.35	±	0.35	0.81	±	0.19#
**Oxidative stress**											
oxLDL	μg/dl	<132	41.22	±	14.48	40.78	±	1.90	35.08	±	13.19
ROM	mg/dl	20–24	30.17	±	4.01	29.95	±	4.79	31.60	±	4.20
PON1	μg/dl	14–38	25.43	±	8.99	51.50	±	18.52*	42.62	±	4.53*
PAT	μmol/l	3080–3920	3867	±	312	4466	±	344*	4376	±	286*
**Hcy metabolism**											
Hcy	μmol/l	5–15	12.26	±	2.75	13.48	±	2.66	14.18	±	4.03
Vitamin B6	μg/l	3.6–18	29.52	±	19.28	51.49	±	19.97*	43.36	±	21.14*
Vitamin B12	pg/ml	200–910	420.67	±	162.83	496.09	±	211.98*	466.70	±	188.43
Folates	ng/ml	>2.5	9.21	±	4.15	11.62	±	5.16	13.62	±	4.10*

*Significantly different from pre-race values, p<0.05

^#^significantly different from intra-race values, p<0.05.

^§^Ht levels significantly decreased after 14 and 24 h of racing as compared to baseline values, indicating the presence of hemodilution throughout the competition. For this reason, all the biochemical parameters were corrected for changes in plasma volume, as previously indicated [7]. Abbreviations: Hcy, homocysteine; HDL, high-density lipoproteins; LDL, low-density lipoproteins; oxLDL, oxidized low-density lipoproteins; PAT, plasma total antioxidant capacity; PON1, paraoxonase 1; ROM, reactive oxygen metabolites; TC, total cholesterol; TG, triglycerides.

The common cholesterol ratios TC/HDL, LDL/HDL, and TG/HDL, already relatively low at the start of the race, reached the lowest values at the finish. Of particular interest is the ratio TG/HDL, which reflects the size and density of LDL: a low ratio is associated with a reduced concentration of smaller and denser LDL molecules, which are more atherogenic in comparison to larger and less dense particles, due to their greater susceptibility to oxidation [25]. In this context, one of the focuses of the study was to evaluate whether the prolonged physical activity could be associated with oxidative modifications of LDL particles, due to ROS production during the competition [5]. Overall, no evidence of greater oxidation of LDL over the 24-h period of physical activity was found (Table 3). The absence of increased oxLDL could be justified by the significant increment of HDL levels during the performance, which led to a concomitant increase of serum PON1 concentration, an antioxidant enzyme associated with HDL [18]. Thanks to its esterase activity, PON1 is able to hydrolyze oxidized lipids, thus protecting against the oxidative modifications of LDL and the subsequent atherosclerotic lesion formation [17].

**Fig 1:**
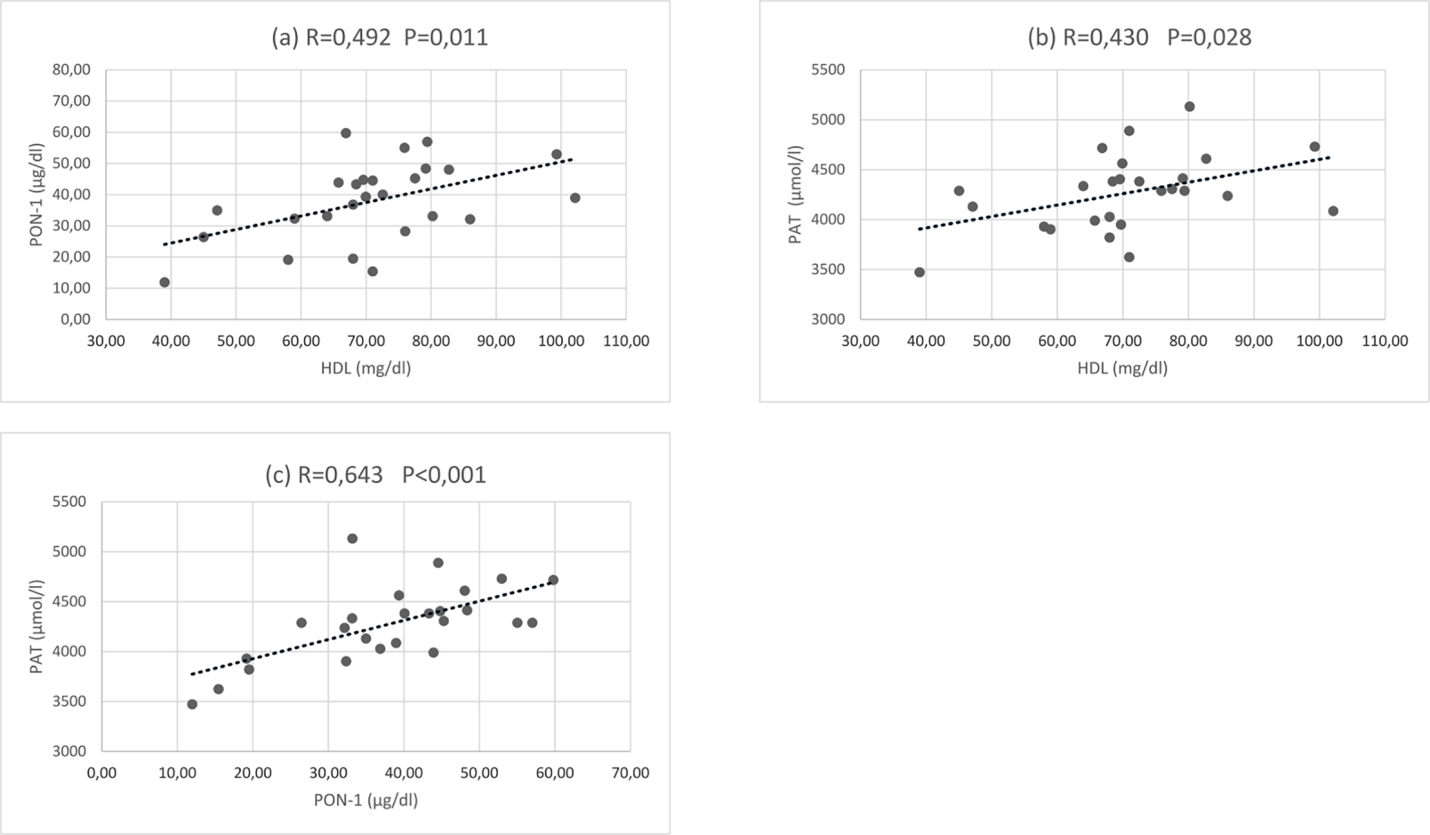
Linear correlations between HDL and PON1 (a), HDL and PAT (b), and PON1 and PAT (c).

As indicated in Fig 1, PON1 concentration positively correlated not only with HDL levels (R = 0.492, P = 0.011), but also with PAT values (R = 0.643, P<0.001), which significantly increased after 14 h of racing, and persisted until the end of the performance. The increment of plasma total antioxidant capacity was already described in other ultraendurance events (100-km, 160-km, and 246-km races) [3, 10, 26], confirming that exercise may activate the endogenous antioxidant mechanisms in response to the long-term aerobic physical activity, thus to counteract the enhanced ROS production [27]. In this context, ROM levels, reflecting the serum content of hydroperoxides as markers of oxidative stress, were assessed for the first time during a 24-h race, revealing that values remained almost unchanged throughout the event (Table 3). Previous studies on oxidative changes during similar ultradistance foot competitions evidenced an increment of other oxidative biomarkers at the end of the race, such as malondialdehyde and F_2_-isoprostanes [3, 10]. Herein, the lack of ROM accumulation might be properly explained by PON1 increase, whose antioxidant activity efficiently degrades hydroperoxides [17]. Accordingly, aerobic exercise was found to increase PON1 levels as an intrinsic antioxidant system adaptation to oxidative stress [14, 28].

Together with oxLDL, elevated serum Hcy concentrations may also contribute to plaque formation and increased cardiovascular risk, through mechanisms involving ROS generation, lipoprotein oxidation and protein homocysteinylation [14, 15]. To date, the effect of exercise on Hcy concentrations remains controversial; indeed, results obtained from several studies are contradictory and sometimes inconclusive, due to differences in methodologies, exercise interventions, and participant characteristics [29]. Herein, the impact of the 24-h run on Hcy levels was investigated for the first time. No evidence was obtained on significant Hcy accumulation during the race (Table 3), presumably justified by the concomitant increments of vitamins B6, B12, and folates, having athletes free access to supplements containing B-group vitamins throughout the entire event (see S1 File). An adequate vitamin B status is critical to maintain optimal Hcy levels [15]; accordingly, Hcy concentration negatively correlated to vitamin B12 levels (R = -0.480, P = 0.018).

## Conclusions

Overall, this study evidenced that the 24-h ultramarathon race was associated with numerous alterations of normal physiological processes, including induction of muscle damage, changes in immune function and increased inflammation. Despite the aerobic overload, the biochemical parameters related to oxidative stress, namely oxLDL and ROM, did not significantly accumulate during the competition, probably due to the favorable increments of PON1 and PAT, which sustained the antioxidant defense system as a mechanism of adaptation. At the same time, Hcy levels remained almost stable throughout the performance. As a conclusion, the present investigation did not specifically evidence a contribution of oxLDL and Hcy in promoting adverse cardiovascular events in 24-h ultramarathon runners; rather, it supported for the first time a protective role of PON1 in contrasting lipoprotein oxidation during the competition. However, the presence of abnormal values of cardiac markers indicates that cardiac stress may occur in such challenging sporting events; therefore, the long-term effects of these alterations, while maintaining the routine practice of prolonged strenuous physical activity, deserve to be carefully monitored.

## Supporting information

S1 File Qualitative information on food and liquid intake. Individual food, liquid and supplement intake during the 24h-race.
(XLSX)

S1 Table Individual data points of Table 1. Individual baseline characteristics and ultramarathon records of the runners which completed the race (n = 9).
(XLSX)

S2 Table Individual data points of Table 2. Individual ultramarathon-induced changes in basic blood count, leukocyte subpopulations, markers of organ function and damage, and inflammation.
(XLSX)

S3 Table Individual data points of Table 3. Individual ultramarathon-induced changes in blood parameters related to metabolic profile, oxidative stress, and Hcy metabolism.
(XLSX)
